# Transcriptomic dissection of the rice – *Burkholderia glumae* interaction

**DOI:** 10.1186/1471-2164-15-755

**Published:** 2014-09-03

**Authors:** Zenaida V Magbanua, Mark Arick, Teresia Buza, Chuan-Yu Hsu, Kurt C Showmaker, Philippe Chouvarine, Peng Deng, Daniel G Peterson, Shien Lu

**Affiliations:** Institute for Genomics, Biocomputing and Biotechnology, Mississippi State University, Mississippi, MS 39762 USA; CVM Basic Science Department, Mississippi State University, Mississippi, MS 39762 USA; Pediatric, Pneumology and Neonatology, Hanover Medical School, Hanover, Lower Saxony, D-30625 Germany; Department of Biochemistry, Molecular Biology, Entomology and Plant Pathology, Mississippi State University, Mississippi, MS 39762 USA

**Keywords:** Bacterial panicle blight, Disease resistance, Next-generation sequencing

## Abstract

**Background:**

Bacterial panicle blight caused by the bacterium *Burkholderia glumae* is an emerging disease of rice in the United States. Not much is known about this disease, the disease cycle or any source of disease resistance. To understand the interaction between rice and *Burkholderia glumae*, we used transcriptomics via next-generation sequencing (RNA-Seq) and bioinformatics to identify differentially expressed transcripts between resistant and susceptible interactions and formulate a model for rice resistance to the disease.

**Results:**

Using inoculated young seedlings as sample tissues, we identified unique transcripts involved with resistance to bacterial panicle blight, including a *PIF*-like ORF1 and verified differential expression of some selected genes using qRT-PCR. These transcripts, which include resistance genes of the NBS-LRR type, kinases, transcription factors, transporters and expressed proteins with functions that are not known, have not been reported in other pathosystems including rice blast or bacterial blight. Further, functional annotation analysis reveals enrichment of defense response and programmed cell death (biological processes); ATP and protein binding (molecular functions); and mitochondrion-related (cell component) transcripts in the resistant interaction.

**Conclusion:**

Taken together, we formulated a model for rice resistance to bacterial panicle blight that involves an activation of previously unknown resistance genes and their activation partners upon challenge with *B. glumae*. Other interesting findings are that 1) though these resistance transcripts were up-regulated upon inoculation in the resistant interaction, some of them were already expressed in the water-inoculated control from the resistant genotype, but not in the water- and bacterium-inoculated samples from the susceptible genotype; 2) rice may have co-opted an ORF that was previously a part of a transposable element to aid in the resistance mechanism; and 3) resistance may have existed immediately prior to rice domestication.

**Electronic supplementary material:**

The online version of this article (doi:10.1186/1471-2164-15-755) contains supplementary material, which is available to authorized users.

## Background

Bacterial panicle blight (BPB) is an emerging disease on rice in the United States [1–4]. BPB was first reported in Japan as the cause of grain rotting and seedling blight on rice [5]. The disease can also cause sheath rot and panicle blight [6]. Diseased panicles are characterized by having florets with a darker base and a reddish-brown margin, and frequently upright due to poor filling. Epidemics of the disease occurred during 1995 and 2000 with yield losses in some fields estimated up to 40% [3, 6]; however, the disease can be worse in years with hot summers [7]. With global temperature on the rise, it is expected that the disease will pose a threat to rice production worldwide [1]. Currently, rice that is highly resistant to the disease is not available commercially and some rice varieties, such as Clearfield 161 showed promising, but not complete resistance to the disease [8]. Major practices to control the disease are use of pathogen free seeds and application of antibiotics [9].

The bacterium *Burkholderia glumae* is the major causal agent of BPB of rice [3]. Pathogenicity and virulence factors of *B. gluma*e include the phytotoxin toxoflavin [10], lipase activity [11] and the motility driven by flagella [12]. Type III effectors that are delivered into host cells by a type III secretion system play critical roles in bacterial pathogenicity and these effectors challenge host resistance mechanisms in many bacterial pathosystems [13, 14]. Thus, mutation in any of the genes encoding a type III secretion system could significantly attenuate the virulence of *B. gluma*e [15]. On the host side, extensive research has been performed on plant resistance against rice blast (caused by the fungus *Magnaporthe grisea*) and bacterial blight (caused by the bacterium *Xanthomonas oryzae* pv. *oryzae*) [16–35]. Although weakly virulent strains of *X. oryzae* pv. *oryzae* used to be found in 1989 [36] bacterial blight has not been identified in the U.S. since then [37]. Because it is caused by a different pathogen, we believe BPB needs to be investigated especially the resistance mechanism against it.

The goal of this research was to understand the rice – *B. glumae* pathosystem using transcriptomics via next-generation sequencing and bioinformatics. Here, we present data that the mechanism for resistance against BPB is independent of resistance mechanisms against other known rice diseases and other novel findings related to this pathosystem.

## Results

### Responses of moderately resistant and susceptible rice varieties to inoculation with *B. glumae*

**Figure 1:**
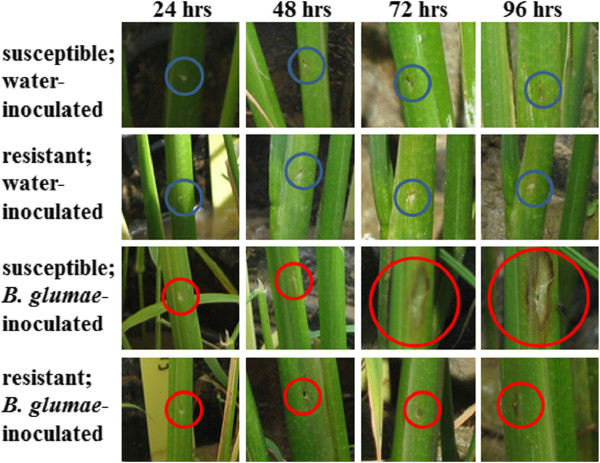
**Comparison of symptoms between a resistant and a susceptible rice.** Symptoms were allowed to develop after 24, 48, 72 and 96 hours on seedlings inoculated with either water or *B. glumae* suspension on a susceptible (CL 151) or a resistant (CL 161) rice. Blue (water) and red (*B. glumae*) circles denote inoculated parts.

Previous studies have shown that rice varieties CL 161 and CL 151 are moderately resistant (R) and susceptible (S) to BPB, respectively [8]. Because we have not identified a source of true resistance, we focused our study on these two genotypes to generate a differential gene expression profile that pertains to the rice – *B. glumae* pathosystem. Although BPB is a disease of the panicle in the U.S., symptoms are also observed in seedlings in other countries [38]. In this study, we were interested in the differential gene expression in young rice plants between an R and an S interaction upon *B. glumae* challenge. We used the *B. glumae* strain 189gr-4 [3] in all inoculations because it has been established for research in BPB. We inoculated young stems and leaf sheaths via injection of 0.5 mL of the inoculum and observed the response 24, 48, 72 and 96 hours after inoculation. After 24 hours, the inoculation marks were visible in all plants and there was no difference between the two genotypes in either the water- or *B. glumae*-inoculated plants (Figure 1). However, after 48 hours, the *B. glumae*-inoculated S genotype plants started to display noticeable browning around the injection sites. The corresponding water-inoculated controls for both genotypes and the *B. glumae*-inoculated R genotype plants showed similar responses at the 24 hour observation (Figure 1). By 72 hours, the browning around the injection sites had increased and had progressed to early stages of sheath rot in the S genotype plants. By contrast, those plants with the R genotype showed only slight browning and drying around the injection sites. The water-inoculated control plants from both genotypes displayed dried areas around them, but not browning (Figure 1). At 96 hours, the necrosis around the injection sites of S genotype plants had spread significantly (Figure 1) and in one plant had extended to most of the entire leaf sheath (not shown). There was not much change from 72 to 96 hours in the water-inoculated controls and an increase in the area of browning in the *B. glumae*-inoculated R genotype plants (Figure 1).

### Illumina libraries and preliminary analysis of Illumina sequence reads

Since expression in the rice - *B. glumae* pathosystem has not been studied, we chose RNA-Seq to conduct a broad account of the interaction. The response showed by the inoculated tissues in the R and S rice genotypes provided a logical progression of disease symptoms and allowed us to choose a time point of study for the transcriptomic analysis. Because the earliest time point that displayed a significant difference in the responses between the two genotypes was at 48 hours, we selected this time point for the transcriptomic analyses. The quality of total RNA was evaluated on non-denaturing agarose gels and a Bioanalyzer. Bioanalyzer outputs ranged from RNA integrity number (RIN) of 7.5 – 9, indicating high quality total RNA was extracted, which was appropriate for any downstream application including RNA-Seq. Likewise, gel pictures showed intact ribosomal RNA bands with minimal smearing that represents the mRNA collected from the samples (Additional file 1). In addition, the amount of RNA shown by the gel pictures was consistent with the Bioanalyzer outputs. Preliminary data from Bowtie [39] and rpkmforgenes.py [40] estimated the total numbers of reads that aligned to the reference genome from each data point and are shown in Table 1. There are 56,986 genes annotated for rice (http://rice.plantbiology.msu.edu/analyses_facts.shtml) and on average, more than 83.58% of the total reads aligned with the rice reference genome. The alignment statistics for each replicate of each sample point are described in Additional file 2.

**Table 1:** **Number of transcripts expressed at each sample point**

Sample point	Expressed transcripts
SW	22,379
SP	24,114
RW	23,706
RP	21,694

S: susceptible; R: resistant; W: water-inoculated; P: pathogen or *B. glumae*-inoculated.

### Differentially expressed transcripts upon inoculation of rice by *B. glumae*

**Figure 2:**
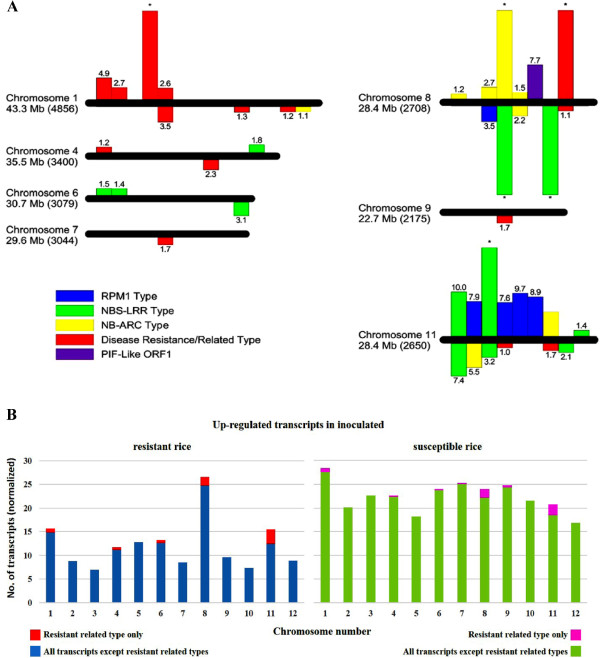
**Chromosomal distribution of differentially expressed transcripts. (A)** The chromosomal locations of resistance-related differentially expressed transcripts in R vs S comparisons are represented. Horizontal bars represent chromosomal axes and are proportional to their lengths while vertical bars represent differentially expressed transcripts. The location of the transcripts on the bars are relative to their chromosomal positions. Above the axes: up-regulated; below the axes: down-regulated; numbers on vertical bars represent log2foldchange while asterisks represent expression only in R (above axes) or S (below axes). The length and the predicted number of genes per chromosome (in parentheses) are indicated [59]. **(B)** The chromosomal locations of all up-regulated expressed transcripts in R and S are shown. To allow for comparison, the number of up-regulated transcripts was normalized by dividing the actual number by the predicted number of genes [59] and multiplying by 1000 to get a whole number CL 161 is R and CL 151 is S.

The program DESeq [41] was used to determine the transcripts that were up-regulated and down-regulated in the R genotype versus S genotype (R vs S) upon inoculation, and in the same genotype upon inoculation compared with the control (water-inoculated). The numbers of transcripts that were differentially expressed using a false discovery rate (FDR) cutoff of 5% between selected sample points are enumerated in Table 2. The differentially expressed transcripts in the R vs S comparison are enumerated in Additional file 3. Upon inoculation, less rice transcripts were up-regulated in the R vs S comparison (456) than those that were down-regulated in the (855). In addition, there were also more transcripts that were up-regulated upon inoculation (compared with the control) of the S genotype (3,780) than those that were up-regulated upon inoculation of the R genotype (2,340). A cursory look at the transcripts indicated that the locus in chromosome 10 where the QTL *qRBS1* was mapped from a previous study [42] was not differentially expressed and that disease resistance and disease resistance related transcripts were co-expressed in both genotypes in the *B. glumae* and water-inoculated controls studied (data not shown). These differentially expressed transcripts from this study were selected and shown in Figure 2A. The disease resistance-type transcripts include NBS-LRR [43–48], NB-ARC [49] and RPM1 [50, 51] classes. In addition, other disease resistance/related type transcripts were also selected and shown in the same figure. These transcripts were only mapped in chromosomes 1, 4, 6, 7, 8, 9 and 11, with greater representation from chromosomes 8 and 11. However, when all differentially expressed transcripts were normalized and analyzed by chromosomal location, a clustering of up-regulated transcripts in chromosomes 8 and 11 was observed in the R vs S comparison (Figure 2B), suggesting a role for these two chromosomes in resistance to BPB. Of interest, a *PIF*-like ORF1 [52–56], mapped in chromosome 8, was co-expressed in the R genotype and with almost no expression in the S genotype, in both control and inoculated samples (Figure 2A). *PIF* is a family of Class 2 transposable element that is widely distributed in plants and some metazoans [55, 57, 58].

**Table 2:** **Number of differentially expressed transcripts between conditions tested**

Sample point	SW	SP	RW	RP
**SW**	NA	3,735	2,538	DC
**SP**	3,780	NA	DC	855
**RW**	2,160	DC	NA	1,840
**RP**	DC	456	2,340	NA

Numbers denote up-regulated transcripts in Row/Column conditions. S: susceptible; R: resistant; W: water-inoculated; P: pathogen or *B. glumae*-inoculated; NA: not applicable DC: did not calculate.

We checked for the expression of known disease resistance genes against blast and bacterial blight in rice such as *Pi-2* [16], *Pi-36* [17], *Pi5-1* [18], *Pi9* [19], *Pib* [20], *Pi-d2* [21], *Pikm1-TS* [22], *Pikm2-TS* [22], *Pikp-2* [23], *Pit* [24], *Pita* [25], *Piz-t* [16], *Xa21* [26, 27], *Xa26* [28, 29], *Xa1* [30], *Xa27* [31], *xa5* [32]; and *xa13* [33–35]. Of these, one putative *xa1* transcript (*LOC_Os02g16330*) was detected in the control S and in the inoculated R conditions, while another (*LOC_Os02g16260*) was shown in the control R. In these three cases, the levels were very low, implying background levels. The rest of the other genes were not expressed in the inoculated R, which suggests that the resistance genes against blast and bacterial blight are not involved in BPB. Further, this suggests that the resistance pathways in rice against *B. glumae* are different from blast and bacterial blight. Of note, these known genes were not expressed in the control R, control or inoculated S either.

### Gene Ontology annotation of differentially expressed transcripts

**Figure 3:**
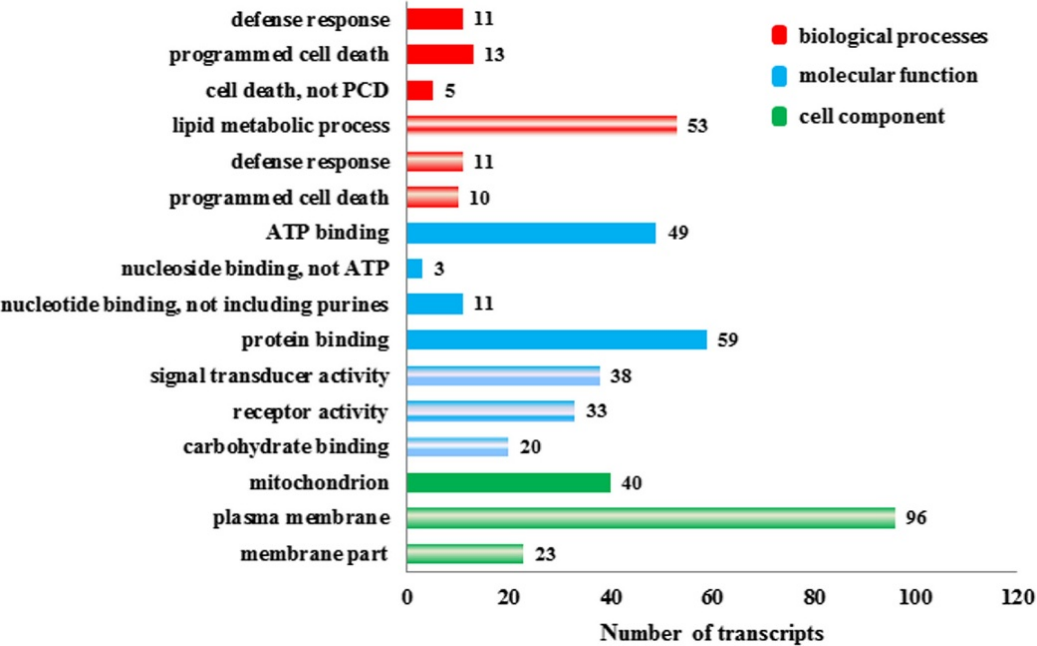
**Functional annotation of up-regulated transcripts.** Gene Ontology was used to functionally annotate transcripts that were up-regulated in rice from R vs S (solid patterns) and S vs R (striped patterns) comparisons were analyzed by Gene Ontology. Number of transcripts that support each annotation are shown in each entry. CL 161 is R and CL 151 is S.

Because the differentially expressed transcripts may provide clues on the resistance mechanism taking place in rice, we used them as input for Gene Ontology (GO) [60]. GO provides functional annotations to transcripts and proteins, which when grouped together according to expression patterns may offer insight into the mechanism of the system being studied. The results are summarized in Figure 3. For transcripts that contribute to biological processes, those involved in programmed cell death and defense response were significantly up-regulated in both the R vs S and S vs R comparisons although they were from different transcripts (loci). In addition, transcripts that play a part in lipid metabolic process were up-regulated in S vs R, while those that contribute to unknown cell death (not programmed) are up-regulated in R vs S. In those transcripts annotated for molecular function and cellular location, we observed a remarkable difference between the up-regulated transcripts in R vs S and S vs R. Transcripts associated with nucleotide binding specifically ATP and protein binding as well as transcripts related to the mitochondrion were up-regulated in R vs S, which suggests that energy production systems in the inoculated R genotype are expressed better that in the inoculated S genotype. By contrast, transcripts involved in carbohydrate binding and signal transducer and receptor activities and those located on plasma membrane and other membrane parts were up-regulated in S vs R.

### Validation of selected differentially expressed transcripts

**Figure 4:**
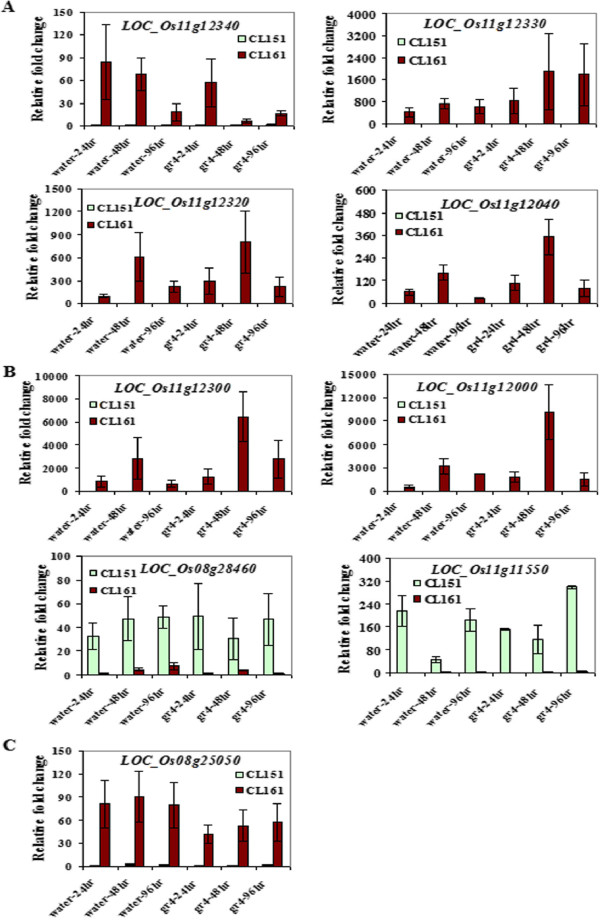
**Validation of differential expression of selected transcripts using qRT-PCR.** Comparative analysis between an R (CL161) and an S (CL151) interaction involving rice and *B. glumae* using quantitative RT-PCR analysis of RPM1 **(A)**, NBS-LRR **(B)** and PIF-like RF1 **(C)** transcripts from rice. Bars represent standard error.

To validate their expression, we selected a few of the disease-resistance related transcripts (RPM1 and NBS-LRR-type) that were differentially expressed with high fold change values and were mapped in either chromosome 11 or 8, and a *PIF*-like ORF1 and measured their expression after 24, 48 and 96 hours of inoculation using quantitative real time PCR (qRT-PCR). Figure 4 shows the summary of the results. Five (three RPM1-type transcripts and two NBS-LRR-type transcripts) out of 21 transcripts tested were only expressed in the R genotype while two (a RPM1-type transcript and the *PIF*-like ORF1 transcript), had minimal expression in the S genotype, but both had high expression in the R (in the control and inoculated conditions). They all were mapped to chromosome 11 except for *PIF*-like ORF1 (mapped to chromosome 8). All seven showed fold change values ranging from 10 to 10,500 (R vs S) in the control and inoculated states, suggesting constitutive expression in the R genotype plants. One transcript (*LOC_Os11g12340*, RPM1-type) showed decreasing expression from 24 to 48 hours upon inoculation after which it showed an increase. In comparison, it showed decreasing expression from 24 to 96 hours in the water control (Figure 4A). Two NBS-LRR-type (*LOC_Os11g12000* and *LOC_Os11g12300)* and two RPM1-type transcripts (*LOC_*Os11g12040 and *LOC_*Os11g12320) appeared to have had a spike in fold change values after 48 hours of inoculation. Likewise, they all displayed higher expression at 48 hours compared with the other time points in the water control but not as high as the 48 hour inoculated time point (Figures 4A and B).

## Discussion

Rice is one of the most studied plants for several reasons, one of which is its economic significance. Major pathogens to rice include *Magnaporthe grisea*, the causative agent of rice blast and *Xanthomonas oryzae* pv. *oryzae*, the causative agent of bacterial blight. Because these diseases inflict rice, extensive research has been done to understand host-pathogen interactions and develop disease management strategies. As a result, most rice resistance genes have been cloned against these diseases (Results Section). In this study, we embarked to understand BPB, a rice disease caused by another pathogen, *B. glumae*. To obtain a broad representation of the interaction between rice and *B. glumae*, we used RNA-Seq to identify transcripts that were differentially expressed between a resistant and a susceptible interactions. With young stems from seedlings as test tissues, we observed symptom development after 24, 48, 72 and 96 hours of inoculation. Stems over panicles were chosen for better distinction of symptoms between a susceptible and a resistant interaction over a short period of time. Reports have shown that symptoms on the panicles usually appear after two weeks of inoculation [3, 4]. At this time period, gene expression may not be indicative of what happens early in the interaction, but earlier time points may not show significant difference in the symptoms in the panicles. In the stem tissues, browning and lesion formation progressed at a remarkable rate in the susceptible genotype, especially when compared to the control and the resistant interaction. However, we found that after 48 hours was the earliest time point that showed the most difference in the responses between the two genotypes. This was our basis for choosing 48 hours post inoculation for the RNA-Seq experiment. We opted for replicates over deeper sequence coverage to provide statistical measures on comparative analysis between any two sample points as we were ultimately interested in possible sources of resistance genes from rice. Haas *et al.* [61] expressed the same sentiment when they said that for some systems, the tradeoff for having replicates rather than sequence depth will provide better biological insight and statistical confidence. We had three biological replications and each replicate was loaded into two lanes. We pooled the reads from two lanes to constitute the read counts for a specific replicate (multiplexed with other samples). The application of the appropriate bioinformatic tools was equally necessary. In this work, we used Bowtie and rpkmforgenes.py script using default parameters to pre-analyze our data, and DESeq to calculate for the differentially expressed transcripts. High-throughput data analysis requires accurate prediction of variability within the dynamic range of values and a suitable error model and DESeq attempts to achieve them by using the negative binomial distribution with the variance and mean linked by local regression. DESeq was preferred over other programs because it provides better statistics for high-throughput data with few numbers of replicates such as RNA-Seq and it addresses the issue of data normalization in a more robust way compared to other available systems [62]. As an initial filter after alignment into the rice genome, we eliminated reads that mapped to more than one locus, rRNAs and other repetitive sequences such as transposable elements. DESeq calculates padj (p-values adjusted for false discovery rate) to correct for multiple testing. We applied an FDR cutoff of 5% and used fold change values of 2 or greater to select for differentially expressed transcripts in the host (rice). These stringent conditions nevertheless generated sufficient transcripts for analysis.

The rice genes that were differentially expressed between the resistant and susceptible interaction were disease resistant-types or related, different enzymes, transcription factors, expressed and hypothetical proteins as well as proteins of unknown function. An enumeration of the transcript-types did not show significant distinction between the two types of interactions. However, a GO analysis, which classifies genes into biological process, molecular function and cellular component, demonstrated a clear distinction between them. In the R interaction, we saw an up-regulation of transcripts that are enriched for defense response, programmed cell death and generalized cell death transcripts under biological process; ATP, nucleoside, nucleotide and protein binding transcripts under molecular function; and mitochondrion supporting transcripts for cell component. By contrast, the S interaction displayed an up-regulation of transcripts enriched in lipid metabolic process, defense response and programmed cell death under biological processes; signal transducer, receptor activities and carbohydrate binding under molecular functions; and plasma membrane and membrane parts under cell component. This side by side comparison of ontologies presented that although disease resistance transcripts and most likely proteins were also up-regulated in the S interaction, other constituents appeared to play important roles in the resistance mechanism. For example, transcripts supporting molecular function and cell component were different between the two interaction types. Of note, a QTL for bacterial seedling rot, another rice disease caused by *B. glumae* is *qRBS1*, was mapped in chromosome 10 [42]. This locus was not differentially expressed in any of the comparisons made, suggesting different pathways for resistance in these two rice diseases even if they were caused by the same pathogen. When we looked closer into specific disease-related transcripts, none of the previously cloned rice genes were differentially expressed, suggesting the known resistance genes were not involved in this interaction. Most were not even expressed at the tested conditions. The differentially expressed transcripts may represent genes that are unique to the rice - *B. glumae* interaction, indicating that resistance of rice to BPB may be conferred by a different set of genes and their roles in the interaction need to be further investigated. Of characterized resistance-related transcripts, those of the NBS-LRR-type and in some cases, sub-families such as NB-ARC and RPM1, were found to be both up-regulated and down-regulated in the R vs S comparison (down-regulation means up-regulation in the S vs R comparison), though actual transcripts associated with each group were not shared. We selected a few of these transcripts to verify their expression using qRT-PCR. All six disease-related transcripts (four RPM1 and two NBS-LRR types) were co-expressed in both the control and inoculated R conditions but not expressed at all in the control or inoculated S. A *PIF*-like ORF1 that is mapped in chromosome 8 follows the same trend. These results suggest that the R genotype maybe keeping a constitutive level of resistance arsenal to help it combat future *B. glumae* attacks.

It has been documented that the nucleotide-binding site or NBS (also NB-ARC) is a conserved domain for ATP binding and hydrolysis and sequences at the amino terminus are required for protein-protein interaction [48, 49]. The leucine rich repeats (LRRs) vary in number and the amino terminal domain seems to regulate activation while the carboxy terminal domain appears to function in recognition [48]. It implies then that where NBS-LRR resistance genes are involved, so does specificity in the interaction. More so, it also suggests the involvement of an effector protein, which initiates the cascade of events that will lead to resistance. RPM1, a type of NBS-LRR resistance gene was originally cloned from Arabidopsis in response to the bacterium *Pseudomonas syringae* [50]. Prior studies had shown that the NBS-LRR gene family is constitutively negatively regulated [63–66] and gets activated in the presence of pathogens through a mechanism that is not clearly understood. However, their activation needs to be precise (in space and time) for resistance to ensue. In addition, earlier studies have demonstrated indirectly that the NBS motif binds to ATP or GTP for activation [48, 49, 64]. If this gene family functions in the same manner in this pathosystem, then processes necessary for their activation should be up-regulated or activated as well. Our GO annotation results suggest an enrichment of ATP binding activities under molecular function, supporting the premise that an activation of NBS type motifs occurs in the R genotype during *B. glumae* challenge. No evidence for NBS activation was shown in the inoculated S genotype, despite the up-regulation of this type motif.

The rice genome has been annotated with more than 500 NBS-LRR-type genes although more than a hundred were predicted to be pseudogenes [46, 47]. Available literature shows that they cluster where mapped [46] and high sequence diversity exists in both the NBS and the LRR domains [47]. It has been proposed that this gene family arose by several independent events of gene duplications all throughout rice evolution [46]. Prior research also demonstrated that diversifying selection has shaped the evolution of the family, giving rise to the diversity that has been observed among its members [47]. Hence, it is conceivable that of those members that are functional, the mechanisms that they provide may not necessarily be similar. Our results showed a clustering of up-regulated transcripts in chromosomes 11 and 8 in the R vs S comparison. Although previous work showed a bias clustering of disease-related genes in chromosomes 11 and 12 [67], our results suggested that resistance against BPB was not a direct result of clustering alone as none of the resistance genes in chromosome 12 were differentially expressed between the conditions tested. Furthermore, there are other loci where resistance genes are clustered, though to a lesser degree [46]. Our results suggest that rice may have utilized the clusters of resistance genes together with another factor/s to devise a resistance mechanism against BPB. These factors may include NBS-LRR activation partners like ATP binding. Another interesting finding was the co-expression in the water- and pathogen-inoculated states, and probably, constitutive expression of a *PIF*-like ORF1 transcript in the R and almost none in the S genotype. Because the filtering method for the reads that we used involved elimination of those mapped to more than one locus and known repeats like rRNA and transposable elements, we can state that the reads only mapped to the *PIF*-like ORF1 transcript in the genotypes that we studied. When we further investigated it from the Rice Genome Annotation Project [68], it showed only one match (located in chromosome 8), suggesting that only one copy exists per haploid genome. P *instability factor* or *PIF* [52–56], a family of Class 2 transposable element is widely distributed in plants and other metazoans [55, 57, 58]. Jiao and Deng [58] performed a genome-wide survey of transcriptional activity of transposable element-related genes in 15 developmental stages and stress conditions in rice and found no expression of *PIF*-like transcripts in their test plants, suggesting that the *PIF*-like ORF1 is not expressed in all rice genotypes. *PIF* has two open reading frames, ORF1 and ORF2, of which ORF2 is most likely the transposase TPase [54]. The function of ORF1 is still unknown, but its predicted protein sequence has significant homology to the Myb/SANT domain. The Myb domain is involved in DNA binding [69] while the SANT domain, although shares a strong homology with Myb sequences, is involved in protein-protein interactions [70]. When we searched for homologs of the transposase in our transcriptomes, we found out that the reads mapped to several loci and were eliminated from the analysis. Whether the homologs were truly repetitive or this was an artifact of high-throughput sequencing analysis remains to be explored and is beyond the scope of this study. Based on the GO result that the R genotype did not show significant (p ≤ 0.05) enrichment for signal transduction, it appears that the *PIF*-like ORF1 may have been recruited to behave as a transcriptional regulator through DNA binding and not as a participant in signal transduction processes in this pathosystem. The transcripts that were constitutively expressed in the R genotype as quantified using qRT-PCR were all mapped in chromosome 11, suggesting that the *PIF*-like ORF1 (chromosome 8) may be acting *in trans* on the genes that it regulates.

All things considered, we propose a resistance mechanism in rice against BPB that existed early in rice domestication and that is not shared with other diseases including rice blast and bacterial blight. This was supported by the recent occurrence of this disease [3] and several observations that we noted in this work that are linked to resistance. We propose that shortly before it is domesticated, encounters between rice and *B. glumae* are limited. The genome of rice along with the prevailing environment at that time may have supported resistance. Specifically, the cluster of resistance genes that include NBS-LRR and related types in chromosomes 11 and 8, the up-regulation of the *PIF*-like ORF1 and the enrichment for ATP binding all contribute to this resistance. Because they are available, rice may have co-opted them as resistance contributors against BPB. The involvement of NBS-LRR-type transcripts and activation partner ATP binding suggests that the resistance mechanism consists of an effector molecule, probably from the pathogen, that is recognized by the host. The effector activates a cascade of events that will eventually lead to resistance in the host. It is possible that the *PIF*-like ORF1 may have been recruited to participate in the activation of the NBS-LRR genes. However, changes in global weather patterns, specifically gradual warming, favored the breaking of the resistance originally held by wild rice species. This is not outrageous as an increase in new or previously insignificant plant diseases caused by pathogens that grow optimally at higher temperatures has been observed with the increase in global temperatures [1, 71]. *B. glumae* is one of these pathogens.

Alternatively, because it may have been a part of a DNA transposon, the *PIF*-like ORF1 may be performing a more active role in the resistance pathway. This remains to be tested but is not a part of this study. Furthermore, we do not exclude that other processes may be occurring in parallel. The list of differentially expressed transcripts includes proteins of unknown functions and other disease related proteins. Their roles in the resistance pathway need to be uncovered in order to paint a complete picture of the resistance mechanism.

## Conclusion

The main objective of this study was to understand the interaction between rice and *B. glumae*, the bacterium that causes bacterial panicle blight, using transcriptomics via next-generation sequencing technology and bioinformatics. This is a timely study of BPB as it is an emerging disease in the rice growing regions of the United States. With our strategy, we were able to provide a model for the resistance mechanism and present hypotheses to be tested. Of note is the hypothesis that resistance existed just prior to rice domestication suggesting that sources of resistance may be found in wild species. A good example is *Xa21*, a resistance gene that confers resistance to rice bacterial blight (caused by the bacterium *X. oryzae* pv. *oryzae*) was originally identified in the wild rice relative *Oryza longistaminata* and introgressed into *Oryza sativa* [43]. We have generated candidate loci that may play major roles in conferring resistance against BPB. Along with phenotypic studies on the response of wild species against BPB, these loci may be used as molecular markers as well as foundation to build evolutionary history studies of this disease. However, in order to complete the model, functional assays need to be performed on these candidate loci/genes and similar studies with time series and on other developmental stages of rice especially the panicle stage need to be carried out.

## Methods

A diagram of the Materials and Methods is given in Additional file 4.

### Generation of samples for RNA-Seq and qRT-PCR

*B. glumae* strain 189gr-4, requested from Dr. Jong Hyun Ham, Louisiana State University, was used to inoculate rice seedlings. Originally isolated from Texas, it is a wild type strain with confirmed pathogenicity on rice seedlings and panicles [3]. To prepare the inoculum, the bacterium was grown on NBY plates [72], incubated at 30°C for 24 h, harvested with a sterile cotton swab and suspended in a vial containing sterile distilled water to OD_420_ of 0.3 (10^8^ CFU/mL) [3].

Clearfield rice varieties CL 151 and CL 161 were used for this study [73]. Five to six seedlings were planted in individual pots grown in a greenhouse at Mississippi State University. Each seedling was injected with 0.5 mL of inoculum into the main stem and greenhouse conditions were maintained at 35-40°C during the day with >75% relative humidity. Water-inoculated plants were used as negative control. Three biological replications were completed for each sample point. Symptom development was observed 24, 48, 72 and 96 hours after inoculation. Inoculated parts were cut 1.0 cm above and below the inoculation point and frozen immediately in liquid nitrogen. Total RNA was extracted from tissues using a modified hot borate method [74] or Trizol reagent (Life Technologies, Carlsbad CA).

### Sequencing using Illumina Hi-Seq

Total RNA was treated with DNAse I (Promega Madison, WI) and subsequently cleaned using the RNeasy Mini Kit (Qiagen, Valencia, CA) as described by the manufacturer. mRNA were selected using primers designed for polyA tails. The selected mRNA was subjected to chemical fragmentation that resulted in sizes ranging from 100–300 bp. cDNA construction and attachment of unique barcodes to each sample ensued (Illumina Inc, San Diego CA). The sequencing libraries with covalently attached unique barcodes were constructed from the cDNA using Illumina chemistry. After RT-PCR, a portion of each library (35ul) was loaded onto a Pippin Size Selection System gel cassette (3%) that was programmed to select the final sequencing library (~148 bp). Sample was eluted in 40ul and concentrated to 10ul using AmpureXP beads (Agencourt). Bioanalyzer, Qubit fluorometer and qPCR were used to quantify them. The sequencing pools were composed of twelve individually bar coded samples. Equimolar amounts (10 μl of 10 nM solution) from each library sample were pooled to constitute the samples that were loaded into a lane in a flowcell. Concentrations measured by using qPCR, which is more sensitive, were used as basis in pooling the samples. Each biological replicate of the samples was loaded into two different Hi-Seq lanes, together with eleven other biological replicates. Sequences of 1 × 50 bp single reads were generated. The raw reads that passed quality control were submitted to NCBI (**SRA SRP015433 BioProject: PRJNA174463**).

### High-throughput sequence analysis

After the reads were grouped into their specific sample points, adaptors and indices were trimmed. Sequence reads from rice were aligned using Bowtie [39] against the most recent version of the Rice Genome available from the Rice Genome Annotation Project [68] using default parameters. The alignment quality threshold is not more than two mismatches within the first 28 bases of the read and the sum of the Phred quality at all mismatches may not exceed 70. Read count transcript expression values of every annotated gene region were calculated using the rpkmforgenes.py script [40]. This created a list of gene IDs, loci where they were mapped and their corresponding read counts for each replicate of each sample point. Using gene IDs as reference, preliminary analysis was performed to eliminate those with zero read count values and those mapped to more than one locus, transposable elements and rRNA. This initial result was used as basis for all succeeding analyses. Pairwise analyses between genotypes within an inoculation state and between inoculation states within a genotype were performed to obtain differential gene expression using DESeq [41]. DESeq utilized read counts from the rpkmforgenes.py output and normalized them initially for the analysis. Consequently, this step created read count values that were not whole numbers. For any two conditions being compared, DESeq accepted the read counts from all replicates from these conditions and processed the read counts to derive the fold change values between the two conditions. The differential expression analysis was performed on gene IDs that mapped to one and only one locus. To determine differential expression, significance was set to a padj value of at most 0.05 and a fold change value of 2 was used as cut-off score. Gene Ontology (GO) annotations for the differentially expressed transcripts were identified by searching the primary GO databases [75–77] using the associated GO retrieving tools [78, 79]. Furthermore, GO enrichment for the differentially expressed transcripts was analyzed using agriGO enrichment tools [79]. Default parameters were used in all cases.

### Validation of differential gene expression using qRT-PCR

The stem tissues from 24-, 48- and 96-hr post-inoculation were collected for qRT-PCR validation with three independent biological replicates per tissue sample per time point. There was a total of 36 tissue samples that included 2 rice genotypes (CL 151 and CL 161) × 2 treatment groups (water control and bacterium inoculated) × 3 time points × 3 biological replicates. Total RNA from these 36 samples were isolated using the hot borate method [74] and treated as described in previous section. The first strand cDNA (RT product) from each sample was synthesized from 1.0 μg of total RNA using random primers in the reverse transcription reaction. Gene-specific primers (Table 3) were designed to span at least one intron and amplify a product between 150 to 300 bp. Multiple alignments of close homologs of each candidate transcript were used to select the primers. The amplicon for each candidate transcript from RT-PCR was cloned into the pGEM-T easy vector (Promega, Madison, WI) and sequenced to confirm the specificity and accuracy of the amplicon. The transcript expression was quantified by qRT-PCR analysis with three technical replications for each sample using Power SYBR Green PCR Master Mix kit and the 7500 Fast Real-Time PCR system (ABI, Applied Biosystems, Foster City, CA). The PCR program was performed with an initial incubation at 95°C for 10 min, followed by 95°C for 15 sec, and 60°C for 1 min for a total of 40 cycles. The dissociation curve analysis was applied after each PCR run to verify the specificity of amplicon and the formation of primer-dimers. The amplicon size of qRT-PCR for each candidate transcript was also confirmed by agarose gel electrophoresis. The comparative Ct (2^-ΔΔCt^) method was used to analyze the data [80, 81]. The *18S* rRNA transcript was used as a control (reference gene).

**Table 3:** **Primers used in this study**

Gene	Primer name	Primer sequence (5′ → 3′)
LOC_Os11g12340	RPM1-L1-F (forward)	5′-GAAGCTCTCAAACCAGAATCAGAGGAG-3′
	RPM1-L1-R (reverse)	5′-TATTCACCCTGTTAAGGCTAAGAAAGAC-3′
LOC_Os11g12330	RPM1-L2-F (forward)	5′-AATCTCAAGCCAAGTTCGGAAAATCTC-3′
	RPM1-L2-R (reverse)	5′-CTCACCCTGTTAAGTTTAAGATAGGTG-3′
LOC_Os11g12320	RPM1-L3-F (forward)	5′-CTTCGAGTGCTCGGGCTACATGTTCC-3′
	RPM1-L3-R (reverse)	5′-CTCTCTCCAGCCCTGCCAGTGAATCG-3′
LOC_Os11g12040	RPM1-L4-F (forward)	5′-CACCAACGTATGTAAGCTCCCAGAATG-3′
	RPM1-L4-R (reverse)	5′-CTGGAGTTCTTCCAAACCTGATAACC-3′
LOC_Os11g12300	NBS-LRR2-F (forward)	5′-AACAAGAATAGAAAAGGAACTGAAGATG-3′
	NBS-LRR2-R (reverse)	5′-CTCCTTTCACAAACTTCTTCATTTTCC-3′
LOC_Os11g12000	NBS-LRR3-F (forward)	5′-AGTGAGCAAGGTAACACAGTGATAACG-3′
	NBS-LRR3-R (reverse)	5′-GTTCAGGGTACCCAATTGTTGGCGTC-3′
LOC_Os08g28460	NBS-LRR7-F (forward)	5′-CCTTATGGCAACACTGAATCAGGGGAC-3′
	NBS-LRR7-R (reverse)	5′-ATGCACATGTGGGAAGCAGCAAGATTC-3′
LOC_Os11g11550	NBS-LRR8-F (forward)	5′-GTCCCAAGTCCAAGCTGATATCAGGC-3′
	NBS-LRR8-R (reverse)	5′-GAGTTCTCATATTGTACACAGAACCGG-3′
LOC_Os08g25050	*PIF*-L-F (forward)	5′-GAGACAATACAAGCTATGGAAGAGCC-3′
	*PIF*-L-R (reverse)	5′-CGCACAACATTGTACCTTCTTCGGTG-3′
18S rRNA	18S rRNA-1 (forward)	5′-GGAATTGACGGAAGGGCACCACCAGGC-3′
	18S rRNA-2 (reverse)	5′-GGACATCTAAGGGCATCACAGACCTG-3′

The sequence of each forward and reverse primer used for each transcript/gene is shown.

### Ethics statement

This research did not involve any human or animal subjects, materials or data and therefore did not require any ethics oversight or approval in these respects.

## Electronic supplementary material

Additional file 1: **RNA extraction from sample tissues.** Representative gels of total RNA extraction before and after DNase treatment (**A** and **B**) and PCR to test DNAse treatment **(C)**. Lanes 1, 2: markers, 3: negative control, 4: positive control, 5 – 10: samples. (PDF 133 KB)

Additional file 2: **Alignment data of reads from each sample point.** The initial analysis of the 1 × 50 bp reads consisted of clustering them into their corresponding sample point, trimming the indices and adaptors and aligning them to the rice reference genome. Each sample point was composed of three biological replicates that were pooled after the initial alignment step. (DOCX 12 KB)

Additional file 3: **Differentially expressed transcripts in the inoculated R over S comparison.** A list of all the transcripts that were differentially expressed in the pairwise comparison between the R and the S genotypes after 48 hours of inoculation with *B. glumae* at </=padj values of 0.05 and fold change (R vs S) cut-off of 2. (XLSX 92 KB)

Additional file 4: **Flow chart of Materials and Methods.** A chart of the procedures performed in this research. (PDF 8 KB)

## Abbreviations

BPB: Bacterial panicle blight
R: Resistant genotype or CL 161
S: Susceptible genotype or CL 151
RIN: RNA integrity number
FDR: Flse discovery rate
GO: Gene ontology.
