# Supplementary figures and images for: Transcriptomic dissection of the rice – Burkholderia glumae interaction
Source: BMC Genomics. 2014 Sep 3;15(1):755. doi: 10.1186/1471-2164-15-755 (PMC4165909; doi:10.1186/1471-2164-15-755)

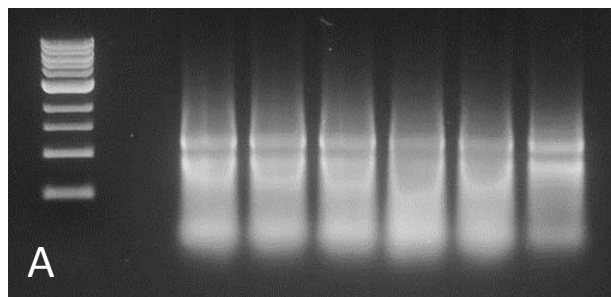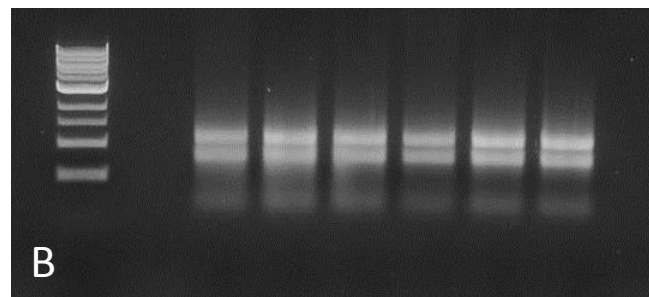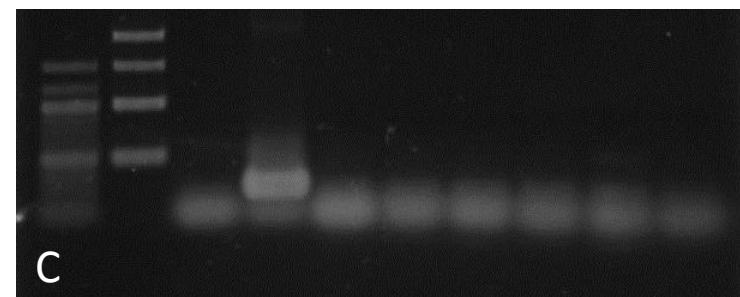

Supplement: Supplementary file 1 — Additional file 1: RNA extraction from sample tissues. Representative gels of total RNA extraction before and after DNase treatment (A and B) and PCR to test DNAse treatment (C). Lanes 1, 2: markers, 3: negative control, 4: positive control, 5 – 10: samples. (PDF 133 KB) [file 12864_2014_6437_MOESM1_ESM.pdf]

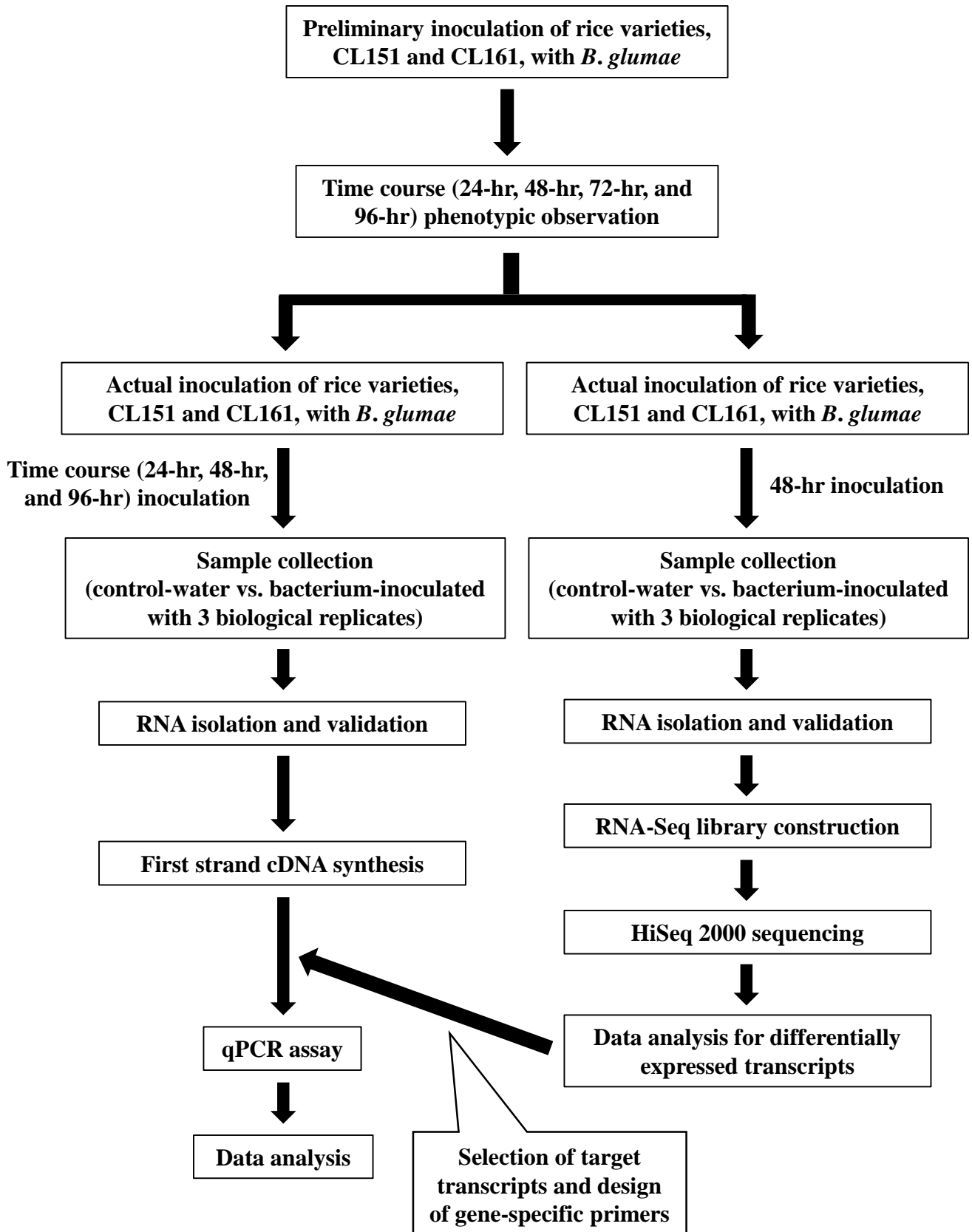

Supplement: Supplementary file 4 — Additional file 4: Flow chart of Materials and Methods. A chart of the procedures performed in this research. (PDF 8 KB) [file 12864_2014_6437_MOESM4_ESM.pdf]
