# Supplementary material for: Transcriptomic dissection of the rice – Burkholderia glumae interaction
Source: BMC Genomics. 2014 Sep 3;15(1):755. doi: 10.1186/1471-2164-15-755 (PMC4165909; doi:10.1186/1471-2164-15-755)
Supplement: Supplementary file 2 — Additional file 2: Alignment data of reads from each sample point. The initial analysis of the 1 × 50 bp reads consisted of clustering them into their corresponding sample point, trimming the indices and adaptors and aligning them to the rice reference genome. Each sample point was composed of three biological replicates that were pooled after the initial alignment step. (DOCX 12 KB) [file 12864_2014_6437_MOESM2_ESM.docx]

| **Data point** | **Number of reads generated** | **Reads that aligned** |
| --- | --- | --- |
| SW, replicate 1 | 15,549,883 | 84.29% |
| SW, replicate 2 | 14,381,198 | 84.43% |
| SW, replicate 3 | 14,848,031 | 84.44% |
| SP, replicate 1 | 12,263,316 | 83.05% |
| SP, replicate 2 | 13,555,356 | 83.03% |
| SP, replicate 3 | 16,070,242 | 82.97% |
| RW, replicate 1 | 21,383,376 | 83.86% |
| RW, replicate 2 | 13,901,773 | 84.34% |
| RW, replicate 3 | 11,744,765 | 83.54% |
| RP, replicate 1 | 14,250,647 | 83.10% |
| RP, replicate 2 | 12,262,894 | 83.56% |
| RP, replicate 3 | 2,568,590 | 82.40% |
